# A review of COVID-19 therapeutics in pregnancy and lactation

**DOI:** 10.1177/1753495X211056211

**Published:** 2022-01-12

**Authors:** Sarah CJ Jorgensen, Najla Tabbara, Lisa Burry

**Affiliations:** 1 Institute of Medical Science, Faculty of Medicine, 7938 University of Toronto, ON, Canada; 2 Department of Pharmacy, 22494 Mount Sinai Hospital, Toronto, ON, Canada; 3 Leslie Dan Faculty of Pharmacy, 7938 University of Toronto, ON, Canada

**Keywords:** Remdesivir, tocilizumab, baricitinib, corticosteroids, bamlanivimab, etesevimab, casirivimab, imdevimab, monoclonal antibodies, lactation, COVID-19, severe acute respiratory syndrome-coronavirus-2

## Abstract

Pregnant people have an elevated risk of severe COVID-19-related complications compared to their non-pregnant counterparts, underscoring the need for safe and effective therapies. In this review, we summarize published data on COVID-19 therapeutics in pregnancy and lactation to help inform clinical decision-making about their use in this population. Although no serious safety signals have been raised for many agents, data clearly have serious limitations and there are many important knowledge gaps about the safety and efficacy of key therapeutics used for COVID-19. Moving forward, diligent follow-up and documentation of outcomes in pregnant people treated with these agents will be essential to advance our understanding. Greater regulatory push and incentives are needed to ensure studies to obtain pregnancy data are expedited.

## Introduction

Since the start of the COVID-19 pandemic, a great deal of progress has been made in our understanding of its effects on maternal and perinatal outcomes. Early data suggested that pregnancy may not be a risk factor for more severe illness. However, growing evidence shows pregnant people have an elevated risk of severe COVID-19-related complications compared to their non-pregnant counterparts.^1–5^ In addition, COVID-19 has been associated with increased preterm birth and neonatal morbidities.^1–5^ Pregnant people from minority ethnic groups have shouldered a disproportionate burden of the negative effects of COVID-19 in pregnancy.^1,4,5^

Despite progress in our understanding of the impact of COVID-19 on pregnant people, there remains limited data on the safety and efficacy of COVID-19 therapies in this special population. The aim of this review is to summarize published data on pregnancy and perinatal outcomes following exposure to antiviral, corticosteroids, biologics, and supportive care agents used for COVID-19. We also summarize available safety data on these agents in breastfeeding, highlight current knowledge gaps, and outline our current clinical practice.

## Antiviral

### Remdesivir

Remdesivir is a nucleoside antiviral drug with broad-spectrum activity against coronaviruses.^6^ It was shown to reduce the time to recovery in hospitalized patients with COVID-19,^7^ but its effect on mortality remains uncertain.^7–11^ Pregnant and breastfeeding individuals were excluded from all remdesivir clinical trials and so there is no direct evidence of efficacy in this population.

Safety data are available from pre-clinical studies, uncontrolled observational studies, and post-marketing surveillance databases.^12–23^ No adverse findings were observed in pre-clinical reproductive toxicity studies with exposures up to 4 times higher than those achieved in humans with recommended dosing.^23^ Placental transfer of remdesivir in humans is unknown. Remdesivir is a prodrug and is rapidly hydrolyzed following intravenous administration,^6^ suggesting the parent drug is unlikely to cross the placenta in clinically important amounts. Major circulating active metabolites have long half-lives, low molecular weights, and high unbound fractions,^6^ suggesting that they may have higher transplacental passage.

We did not identify data describing remdesivir pharmacokinetics in pregnant people. Simulation studies suggest pregnancy-related increases in glomerular filtration rate and renal tubular secretion may increase the elimination of active metabolites.^24^ Additionally, pregnancy-associated changes in plasma protein binding and concentrations may increase the unbound concentrations of remdesivir and its metabolites, which may, in turn, lead to more rapid clearance.^24^ The clinical implications of these potential pharmacokinetic changes are not known.

Published clinical experience with remdesivir in pregnancy outside of clinical trials remains limited. The 2 largest reports are the Gilead Global Safety Database (*n* = 156), which included pregnant Ebola virus infection and COVID-19 patients, and the COVID-19 compassionate use program (*n* = 67).^13,22^ Among those with known pregnancy outcomes in the Safety Database there were 33 live births and 13 adverse pregnancy outcomes (7 spontaneous fetal loss, 2 induced abortions, and 4 stillbirths).^22^ All individuals that experienced adverse pregnancy outcomes were critically ill and required invasive mechanical ventilation within 24 h of remdesivir initiation. Five cases of congenital abnormalities were identified; remdesivir exposure occurred after the first trimester in all cases.^22^

The compassionate use program included a high proportion of critically ill pregnant people: 67% were admitted to the intensive care unit and 40% received invasive mechanical ventilation.^13^ The median gestational age was 28 (range 14–39) weeks, with no first trimester exposures. Overall, 93% recovered within 28 days. Pregnant people not requiring invasive mechanical ventilation had the highest rates of recovery (98%) and the shortest median time to recovery (5 days). Eighteen (69%) neonates were delivered preterm; no neonatal deaths occurred during the observation period and no congenital abnormalities were reported (M. Das, personal communication, 3 May 2021). There was 1 intrauterine death at 17 weeks of gestation in a patient with a serious concurrent bacterial infection and 1 maternal death after delivery, which was attributed to COVID-19.^13^

In terms of other adverse effects, nearly 40% of individuals enrolled in the compassionate use program experienced treatment-emergent graded liver enzyme abnormalities, but grade 3 abnormalities were uncommon.^13^ These liver enzyme abnormalities might be related to remdesivir, COVID-19, other pregnancy-related (e.g. preeclampsia), or unrelated causes.

There are no data on remdesivir in breastfeeding. Since remdesivir has poor oral bioavailability,^6^ infants are unlikely to absorb clinically important amounts from breastmilk. Systemic exposure to metabolites, however, could result from breastfeeding. Reassuringly, a small number of infants have received remdesivir for the treatment of Ebola virus infection or COVID-19 and no adverse effects were documented.^25,26^

Based on the data discussed above, our current practice with remdesivir in pregnant and breastfeeding individuals with COVID-19 is similar to the non-pregnant patient population. We offer it to non-critically ill patients requiring supplemental oxygen if no contraindications are present. We emphasize the lack of safety data for first-trimester exposure during discussions with patients in early pregnancy or during pre-conception care.

### Corticosteroids

Dexamethasone was shown to decrease mortality in hospitalized individuals with COVID-19 requiring oxygen therapy in the RECOVERY trial.^27^ Meta-analyses of 8 randomized controlled trials (RCTs) of corticosteroids suggest this may be a class effect, although the evidence is most certain for dexamethasone and to a lesser extent, hydrocortisone.^28,29^ Pregnant people were excluded from these RCTs, except for REMAP-CAP (number of pregnant patients enrolled not reported)^30^ and RECOVERY (*n* = 4, 0.06% of participants).^27^ Prednisolone or hydrocortisone were recommended as alternatives to dexamethasone for pregnant people enrolled in RECOVERY.^27^

There is limited data on corticosteroid pharmacokinetics during pregnancy. Corticosteroids are highly bound to albumin and corticosteroid-binding globin (CBG), both of which change during pregnancy. Increases in CBG have been linked to decreased unbound fractions of prednisolone and prednisone during pregnancy.^31^ Oral clearance of total prednisolone appears to be increased in pregnancy, but oral clearance of unbound prednisolone remains unchanged.^31^ At this time there are no data to suggest corticosteroid dose changes are needed during pregnancy based on pharmacokinetics alone.

The choice of corticosteroid for pregnant people has traditionally depended upon whether treatment was intended for the mother or the fetus. Hydrocortisone, methylprednisolone, prednisolone, and prednisone are extensively metabolized to inactive metabolites by placental enzymes.^32^ Active retrograde placental transport further limits fetal uptake to ∼10% of maternal exposure.^33^ For these reasons, prednisolone and other short-acting corticosteroids are preferred to treat chronic and acute maternal conditions.^34^

By contrast, when treatment is for the fetus, corticosteroids that have greater transplacental passage are preferred. Synthetic fluorinated corticosteroids, such as dexamethasone and betamethasone, are poorly metabolized by placental enzymes and achieve similar concentrations in maternal and fetal circulation.^32^ This property, coupled with minimal mineralocorticoid activity, drives the recommendation to use antenatal dexamethasone or betamethasone to promote fetal lung maturation.^35^

The association between corticosteroids and adverse pregnancy outcomes has been extensively studied with some studies documenting associations with intrauterine growth restriction, congenital malformations, preterm birth, gestational diabetes, and pre-eclampsia.^34,36^ However, evaluating the safety of corticosteroids in pregnancy is challenging because studies are often confounded by multiple concomitant medications, including potential teratogens, and high-risk pregnancies are typically over-represented, particularly for methylprednisolone, which is often used as rescue therapy or the fluorinated corticosteroids, which, as mentioned above, are used to accelerate fetal lung development.^34,36^ Most studies have not adequately accounted for treatment indication or disease severity.

Data suggests that rates of preterm births and low birth weights are not increased following maternal prednisolone administration.^34^ It is difficult to draw conclusions on the effects of dexamethasone or betamethasone on these outcomes because of the confounders described above.^34^ With regard to congenital malformations following prednisolone exposure, concomitant teratogenic drug exposure with medications, such as mycophenolate mofetil and methotrexate, was implicated in many cases.^34^ Patent ductus arteriosus, blindness, and deafness have been reported with fluorinated corticosteroids, but authors did not deem them to be attributable to the steroid therapy, instead attributing these to the underlying conditions the steroids were treating.^34^ Older studies found small associations between corticosteroids in early pregnancy and orofacial cleft palate,^37,38^ but more recent ones have not,^39–41^ and the current consensus is that corticosteroids do not increase the incidence of orofacial cleft palate above baseline.^34,39^

Amounts of corticosteroids in breastmilk are low and they are considered safe if used for short durations while breastfeeding.^42^ There is limited data on dexamethasone during breastfeeding.^42^ High-dose corticosteroids may temporarily decrease milk supply.

With regard to our local practice, we favor the short-acting corticosteroids, such as methylprednisolone and prednisolone, outside of the antenatal corticosteroid indication, to limit fetal exposure. Efficacy data for methylprednisolone is extrapolated from the acute respiratory distress syndrome literature. We acknowledge that it is unclear if the benefit demonstrated with dexamethasone in RECOVERY is truly a class effect and different approaches are also reasonable.^43^

## Biologics

### Tocilizumab

Tocilizumab is a recombinant humanized monoclonal immunoglobulin G1 (IgG1) antibody that blocks interleukin-6 (IL-6) binding to its receptor, reducing downstream inflammatory signaling.^44^ In two landmark RCTs, tocilizumab reduced mortality in hospitalized adults with severe or critical COVID-19.^45,46^ Of the nine RCTs that evaluated tocilizumab for COVID-19,^45–53^ only the RECOVERY trial permitted enrollment of pregnant people (*n* = 10, 0.2% of participants).^45^ Specific maternal and neonatal outcomes were not reported.

Transplacental transport of IgG is thought to be very low during the first trimester of pregnancy, but it increases steadily after week 13, reaching a peak in the third trimester.^54^ Fetal tocilizumab exposure is therefore likely negligible during the critical period of organogenesis. Pre-clinical reproductive toxicology studies in monkeys support this with no evidence of teratogenicity when tocilizumab was administration in the first trimester.^44^ However, there were dose-related increases in the incidence of abortion or embryo-fetal death at higher relative exposures.^44^ Additionally, in tocilizumab pre-/post-natal mice experiments, offspring showed laboratory indicators of mild immunosuppression.^44^

The pharmacokinetics of tocilizumab in pregnancy have not been characterized. Due to their large size and hydrophilicity, monoclonal antibodies (mAbs) reside almost exclusively in the blood plasma and extracellular fluid.^55^ The increase in blood volume associated with pregnancy^56^ may reduce tocilizumab concentrations. In addition, mAbs are primarily eliminated by intracellular degradation after target binding, and to a lesser degree by proteolytic catabolism.^55^ The former is related to target expression (i.e. IL-6 receptor expression), which is ∼40% higher in pregnant people compared to non-pregnant people,^57^ suggesting tocilizumab may be eliminated more rapidly in pregnancy. Tocilizumab is dosed based on total body weight; the most appropriate body weight descriptor for tocilizumab dosing in pregnancy is uncertain (pre-pregnancy vs. actual body weight). Larger doses or additional doses might be required during pregnancy to match exposures achieved in non-pregnant populations.

Clinical data on tocilizumab use in pregnant people is available from the Roche Global Safety Database (*n* = 288)^58^ and the European League against Rheumatism (EULAR) task force (*n* = 218).^59^ In the Global Safety database, over 90% of cases were for rheumatoid arthritis and most received their last dose pre-conception or early in the first trimester, when tocilizumab would not be expected to cross the placenta.^58^ Of the pregnant people followed prospectively (*n* = 180), there was no increase in congenital malformations compared to baseline rates in the general population (tocilizumab 4.5%; population 3.0–4.0%).^58^ These data are consistent with the EULAR report, which detected congenital malformations in 3.9% of newborns.^59^ Spontaneous fetal losses were higher than in the general population (15–20%) in both the Safety Database (21.7%) and the EULAR report (21.6%).^58,59^ Concomitant methotrexate, which was prescribed to ∼20% of patients, may have contributed to these outcomes.^58,60^ The rate of prematurity was also increased (31.1%) compared to the background rate (15–20%).^58^

In total, 17 cases whose outcomes were captured in the Safety Database continued or resumed tocilizumab beyond the first trimester.^58^ All cases gave birth to live infants and in prospective cases (*n* = 11), the median gestational age at birth was 36 weeks and 4 days, that is, approximately half were preterm deliveries. A small number of patients in other reports received tocilizumab beyond the first trimester, but outcomes were generally not reported separately.^61,62^

There have been three reports of tocilizumab use in pregnant people with COVID-19.^19,63,64^ Tocilizumab was often administered in the third trimester, many patients were critically ill, and a minority of patients received corticosteroids.^19,63,64^ In the largest series from Spain (*n* = 12), all pregnancies resulted in live births, but most had limited neonatal follow-up.^63^ There was one case of maternal cytomegalovirus (CMV) reactivation and subsequent congenital CMV infection.^63^

Maternal IgG1 does not transfer well into breastmilk although concentrations are higher in the colostrum from mothers of preterm infants.^65,66^ Furthermore, IgG oral bioavailability is low due to degradation in the infant digestive tract.^65,66^ In keeping with these general properties, tocilizumab is excreted into breastmilk, reaching peak levels 3 to 5 days after dosing, but the transfer is low with trough milk-to-serum ratios less than 0.0015.^62,67,68^ No adverse effects were observed in reports of breastfed infants whose mothers were treated with tocilizumab.^62,67,68^

Based on the literature above, our practice with tocilizumab in pregnant and breastfeeding individuals with COVID-19 is similar to the non-pregnant population. We consider it a treatment option for non-critically ill patients with worsening respiratory status despite 24 to 48 h of corticosteroids ± remdesivir, as well as for critically ill individuals receiving corticosteroid therapy who are within 14 days of admission if no contraindications are present. We emphasize the limited data on safety in the second and third trimester and suggest live vaccines be delayed to 6 months of age if the infant was exposed to tocilizumab in utero.^69^

### Baricitinib

Baricitinib is an orally administered, Janus-associated kinase (JAK)-inhibitor that interrupts the signaling of multiple cytokines implicated in COVID-19 immunopathology.^70^ It may also have antiviral activity by blocking viral cell entry and suppressing type I interferon-driven angiotensin-converting-enzyme-2 upregulation.^70^ It was shown to accelerate time to recovery in hospitalized patients with COVID-19 when added to remdesivir and more recently, to decrease mortality when added to corticosteroids.^71,72^ Both studies excluded pregnant and breastfeeding people, and pregnancy is an exclusion criterion in the ongoing evaluation of baricitinib in the RECOVERY trial.

Extensive transplacental passage was demonstrated in animal models,^73^ and although there are no human data, as a small molecule, baricitinib is expected to cross the human placenta from the first trimester onward. In pre-clinical animal reproductive toxicology studies, baricitinib was teratogenic and feticidal, although at exposures many-fold higher than those achieved in humans at the maximum recommended dose.^73^

Pharmacokinetics of baricitinib in pregnancy have not been reported. It is rapidly absorbed after oral administration^70^; delayed gastric emptying associated with pregnancy may delay the time to peak concentration but total oral bioavailability is likely not altered. Baricitinib has moderate plasma protein binding and is predominantly cleared by renal elimination.^70^ Pregnancy-associated changes in protein binding and glomerular filtration may lead to more rapid elimination. Only a small fraction of baricitinib is metabolized by cytochrome P450 enzymes, however, it is a substrate of several drug transporters,^70^ the expression or function of which may be altered during pregnancy. Although it is difficult to predict, larger doses may be required during pregnancy to exposure match non-pregnant patient populations.

Clinical data on baricitinib in pregnancy are limited. Twelve women became pregnant while exposed to baricitinib in the clinical trials program for rheumatoid arthritis.^74^ Of these, five resulted in live births with no fetal abnormalities, four resulted in spontaneous fetal loss, one was electively terminated, and two pregnancies were ongoing at the time of the report.^74^ A case report described a woman treated with baricitinib for rheumatoid arthritis from conception to 17 weeks of gestation.^75^ A healthy infant was born at 38 weeks. No perinatal infections occurred and at 9 months the baby had normal growth or psychomotor development.^75^ Tofacitinib is another JAK-inhibitor approved several years before baricitinib. Although data are still limited, the outcomes of at least 60 pregnancies have been reported and adverse pregnancy outcomes do not appear to be increased above expected background rates.^76^

Similar to limited pregnancy data, no human studies have reported pharmacokinetics or safety of baricitinib or other JAK-inhibitors in lactation, but as a small molecule it is likely to be present in breastmilk and baricitinib was detected in the milk of lactating rats at exposures ∼45-fold higher than corresponding plasma exposures.^73^

We do not currently use baricitinib in non-pregnant or pregnant individuals COVID-19. The thrombotic risk that JAK-inhibitors carry is also concerning in pregnant people who are already at heightened risk of thrombotic complications.^70^

### Anti-severe acute respiratory syndrome-coronavirus-2 monoclonal antibodies

Three anti-severe acute respiratory syndrome-coronavirus-2 mAb (anti-SARS-CoV-2 mAb) products directed against the SARS-CoV-2 spike protein have been evaluated for the treatment of COVID-19 in phase III trials: bamlanivimab plus etesevimab, casirivimab plus imdevimab, and sotrovimab.^77–79^ All reduced the composite of COVID-19-related hospitalizations (or all-cause hospitalizations for sotrovimab) and all-cause death in high-risk outpatients with mild to moderate COVID-19.^77–79^ Preliminary data suggest casirivimab plus imdevimab may also reduce the risk of SARS-CoV-2 infection in household contacts of infected individuals and decrease mortality amongst hospitalized patients with COVID-19 who are seronegative at baseline.^80,81^ Pregnant people were excluded from all clinical trials of these products except for the casirivimab plus imdevimab platform of RECOVERY.^80^ In total, 25 hospitalized pregnant individuals were enrolled in RECOVERY (0.3% of participants); specific maternal and perinatal outcomes have not been reported, however.^80^ In addition, enrollment of pregnant people is planned for future platforms of the casirivimab plus imdevimab outpatient study.^77^

Non-clinical reproductive toxicity studies have not yet been completed for any of the anti-SARS-CoV-2 mAbs. As IgG1, they would be expected to cross the placenta in the second and third trimesters (see tocilizumab),^54^ but it is unknown if this confers any benefit or risk to the developing fetus.

Real-world experience with anti-SARS-CoV-2 mAbs in pregnant patients was recently reported in a small case series. Four pregnant people (gestational age 11 to 32 weeks), all with additional risk factors for severe disease, received intravenous casirivimab plus imdevimab for mild or moderate COVID-19 as outpatients. None of the m progressed to severe disease or required additional COVID-19-related medical visits or hospitalizations. At the time of the report, two pregnancies ended in the delivery of healthy neonates at 36 and 37 weeks of gestation; two pregnancies were ongoing.^82^

There are no data on the use of these mAbs in breastfeeding. As IgG1, their transfer into breastmilk is expected to be low and they likely undergo degradation in the infant digestive tract,^65,66^ however, this requires confirmation.

We encourage pregnant people to consider participating in clinical trials of anti-SARS-CoV-2 mAbs if they are eligible. For centers that are providing these therapies to outpatients, we agree with guidelines recommending that pregnancy be considered a risk factor for progression and that pregnant people be offered mAb therapy with diligent follow-up on maternal and perinatal outcomes.^84^ We do not consider anti-SARS-CoV-2 mAbs to be a contraindication to breastfeeding.

## Supportive care agents

### Prophylactic anticoagulants

The prothrombotic state of pregnancy coupled with COVID-19-associated immunothrombosis places pregnant people with COVID-19 at high risk for thrombotic complications.^84,85^ Consensus guidelines for the prevention and management of COVID-19-associated coagulopathy in pregnancy have been released by several expert groups, but high-quality evidence is lacking and recommendations are inconsistent.^84–88^ Our approach to the initiation and duration of prophylactic anticoagulation in the setting of pregnancy and COVID-19 is highly individualized. Our decision-making is based upon patient-specific thrombotic risk factors, severity of COVID-19 infection, organ dysfunction, bleeding risk, inpatient versus outpatient management, and timing of delivery (Table 1^84–89^)

**Table 1.** Thromboprophylaxis in pregnancy and lactation.^84–89^

Anticoagulant	Antepartum considerations	Peripartum considerations	Postpartum considerations	Usual dose
Unfractionated heparin	No placental transfer If close to delivery, UFH may be preferred Use pre-filled syringes or preservative free formulations (avoid preparations containing benzyl alcohol as this preservative has been associated with gasping syndrome/fatal toxic syndrome in premature infants)	Check PTT/aPTT before neuraxial analgesia Can perform neuraxial analgesia 4-6 hours after last dose Can administer prophylaxis 1 hour after neuraxial analgesia	Can initiate 1 hour after neuraxial analgesia or catheter removal (if adequate hemostasis) Lactation: Compatible Infant monitoring: - Rare skin bruising - Blood in urine, emesis, or stool	5,000 units SC every 8-12 hours
Low molecular weight heparin Enoxaparin Dalteparin Tinzaparin		Can perform neuraxial analgesia 10-12 hours after last dose Can administer prophylaxis 4-6 hours after neuraxial analgesia	Can initiate 12 hours after neuraxial analgesia or 4 hours after catheter removal, whichever is greater (if adequate hemostasis) Lactation: Compatible Infant monitoring: - Rare skin bruising - Blood in urine, emesis, or stool	Enoxaparin* 40 mg SC every 24 hours Dalteparin* 2,500-5,000 units SC every 24 hours Tinzaparin* 3,500-4,500 units SC every 24 hours
Heparinoid Danaparoid	Pregnancy considerations: Reserved for pregnant people with allergies or adverse reactions to unfractionated and low molecular weight heparins. Danaparoid is the preferred anticoagulant in pregnant people with HIT. Lactation: Probably compatible.			
Factor Xa inhibitor Fondaparinux	Pregnancy considerations: Reserved for pregnant people with allergies or adverse reactions to UFH and LMWH who cannot receive danaparoid. Lactation: Probably compatible.			
Vitamin K antagonist Warfarin	Pregnancy considerations: Contraindicated in pregnancy (except pregnant people with mechanical heart valves or if alternatives not available/appropriate, requiring close monitoring and tailored pharmacotherapy by a specialist) due to teratogenic and fetal toxicity risk. Lactation: Probably compatible.			
DOAC (thrombin inhibitor) Dabigatran	Pregnancy considerations: Not recommended for use in pregnant people due to insufficient safety data and potential harm to maternal and fetal conditions. Lactation: Not recommended.			
DOAC (Factor Xa inhibitor) Rivaroxaban Apixaban Edoxaban				

*Requires dose adjustment based on renal function; higher doses may be considered in obese patients

aPTT: activated partial thromboplastin time; DOAC: direct oral anticoagulant; HIT: heparin-induced thrombocytopenia, LMWH: low molecular weight heparin; PTT: partial thromboplastin time; SC: subcutaneous; UFH: unfractionated heparin

### Antipyretics

Maternal hyperthermia and fever have been linked to an increased risk of embryonic death, abortion and a wide range of structural and functional birth defects.^90^ Because of this, fever in pregnancy is typically treated aggressively with antipyretics and external cooling.^91^ Fever is among the most common symptoms in pregnant people with COVID-19, reported in up to 40% of those who test positive for SARS-CoV-2.^1^

Paracetamol is the preferred antipyretic in both pregnancy and lactation.^42,89^ Nonsteroidal anti-inflammatory drugs (NSAIDs) are generally avoided for fever management, particularly in the third trimester, due to an associated with increased risk of premature closure of the ductus arteriosus and oligohydramnios.^89^ Use earlier in pregnancy has been linked to elevated rates of miscarriage, structural cardiovascular defects, and multiple other congenital anomalies, although data are limited by confounding.^89^ It should be noted that NSAIDs are still recommended for specific maternal indications (i.e. low dose acetylsalicylic acid for the prevention of preterm preeclampsia and indomethacin as a tocolytic) and they are an acceptable option for postpartum analgesia.^92^ Very low levels are detected in breastmilk and NSAIDs are considered safe in this setting.^42^ Initial concerns that NSAID use could result in increased disease severity in patients with COVID-19 have been allayed by data showing no association with COVID-19-related complications.^93^

## Conclusions

More data are clearly needed on the efficacy, safety, teratogenicity, and pharmacokinetics of drugs and biologics for pregnant and breastfeeding people with COVID-19. New agents are often licensed despite little information on key characteristics such as transplacental passage and drug labeling is unhelpful in informing clinical decisions for pregnant and breastfeeding people. Even for agents that have been in clinical use for decades, data are sparse. This may have contributed to the underutilization of potentially beneficial therapies and vaccines in past epidemics and this pattern is evident during COVID-19 with data from the UKOSS/ISARIC/CO-CIN showing less than 10% of pregnant individuals with COVID-19 received corticosteroids for maternal indications *after* the release of RECOVERY results.^3^

To reduce concerns about the off-label use of therapies in pregnancy, it is essential that pregnant people be included in clinical trials. There was an extensive experience with corticosteroids in pregnancy before the pandemic and limited data on remdesivir and tocilizumab suggested low safety concerns.^26,58,94^ Despite this, pregnant people were systematically excluded from almost all COVID-19 RCTs with these agents and when they were eligible, enrollment was very low. This raises the question of whether more regulatory push and incentives are required to ensure studies to obtain pregnancy data are expedited, similar to processes for pediatric licensing. While the data summarized in this review can serve as a basis for shared-decision making when treating pregnant and breastfeeding people with COVID-19, more high-quality data are needed to ensure their health, and the health of their future offspring, are optimized.
